# Swedish translation and content evaluation of the Empowerment Audiology Questionnaire (EmpAQ—15)

**DOI:** 10.1186/s41687-024-00819-4

**Published:** 2024-12-17

**Authors:** Josefina Larsson, Elin Karlsson

**Affiliations:** 1 ORCA Europe, WS Audiology, Björns Trädgårdsgränd 1, Stockholm, 116 21 Sweden; 2 Audiological Research Centre, Faculty of Medicine, and Health, Örebro University, Örebro, Sweden; 3 School of Health sciences, Faculty of Medicine, and Health, Örebro University, Örebro, Sweden

**Keywords:** Content validity, Empowerment, Hearing loss, Outcome measure, Translation, Qualitative research

## Abstract

**Objective:**

Translating the newly developed Empowerment Audiology Questionnaire, EmpAQ-15 to Swedish, and performing content validation on the Swedish version.

**Design:**

Best-practice principles using forward and back translations which were revised by a committee prior to field testing. Field testing was conducted by cognitive interviews with hearing-aid users talking through and rating the items in the translated questionnaire. Content validation was assessed by examining equivalence, accessibility, acceptability, comprehensiveness, and relevance of interview data. Questionnaire introduction and scoring instructions were evaluated by Swedish audiologists.

**Study sample:**

Ten adult native speaking Swedish hearing aid users, recruited with purposive sampling. Maximum variation based on age, gender, hearing aid usage, and degree of hearing loss. Seven Swedish audiologists assessing instructions for result calculations.

**Results:**

The conceptual equivalence between the Swedish translation and the English original questionnaire was judged to be high overall. The instructions and majority of items were experienced as accessible, acceptable, comprehensive, and relevant. The audiologists showed that they could follow scoring instructions and reason about the results.

**Conclusions:**

This content validity study was the first step towards a Swedish version of a self- report measure of Empowerment for people with hearing loss.

**Supplementary Information:**

The online version contains supplementary material available at 10.1186/s41687-024-00819-4.

## Abstract (fr)

There is a need for using measurement tools that work outside of an English language context. Questionnaires that use best-practice translation and that incorporates solid validation methodology, including language-specific cultural considerations will likely function better when applied in clinical contexts. In Sweden there is a call for working more patient-centered and within a hearing healthcare context the concept of empowerment is one way of enabling this. Empowerment in the context of hearing challenges involves acquiring knowledge, skills and strategies to enhance self-efficacy, participation, and control over hearing solutions and daily life. This study describes the best-practice translation, cultural adaptation, and content validation process of the newly developed and validated Empowerment Audiology Questionnaire, EmpAQ. From analysis of interview data from hearing aid users that completed and discussed the translated questionnaire we learned that the translation matched the original questionnaire. The instructions and majority of items were experienced as accessible, acceptable, comprehensive, and relevant overall. This is a crucial step toward having a Swedish version of EmpAQ to assess empowerment in people with hearing loss.

## Introduction

There has been a shift in the focus of audiology services embracing a more holistic perspective rather than device-centric, with person centered care driving this change [1]. Within person centered care, the patient is seen from a multidimensional perspective recognizing their active role in their own treatment process. Having a person-centered care focus within health care increases both satisfaction and adherence to treatment [2–4].

Empowerment is a concept that for different fields (social, political, educational, psychological), and for different contexts has various definitions. A commonality within the context of healthcare, is that patient empowerment definitions tend to focus on individuals’ ability to have mastery over their own health and healthcare [5, 6]. Within psychology, Zimmerman’s seminal work defines empowerment as the process of enabling patients to gain mastery over an issue of concern to them, from which the outcome is a feeling of control in the given situation. Zimmerman provide a framework for empowerment comprising five dimensions: knowledge, participation, skills, control, and self-efficacy [7]. Recently, based on Zimmerman’s framework, empowerment was conceptualized within the context of hearing as the process by which individuals with hearing-related challenges acquire and use knowledge, skills and strategies and increase self-efficacy, participation and feeling of control over hearing health care, hearing solutions and everyday life [8].

About 20% of the global population has some level of hearing loss [9]. A hearing loss can be congenital (e.g., from genetical syndromes or infections during pregnancy) or acquired (e.g., noise induced, infections, trauma, or age-related hearing loss). Hearing loss becomes increasingly common with age. By the age of 80, 40% of people experience hearing loss, and by the age of 90, this figure rises to 60% [9]. Today, hearing loss is estimated to be the world’s fourth largest reason for disability [10], being a condition that often leads to frustration, loneliness, and isolation due to the lack of ability to communicate [11]. These factors, alone and combined, can have a significant impact on the individual’s quality of life [12, 13].

Hearing rehabilitation (i.e., provision of measures such as hearing aids or cochlear implants, auditory training, communication strategies, counselling, and support, including empowerment), is important to alleviate the consequences of hearing loss. Empowering a person with knowledge, skills, and resources, enhances their ability to communicate, engage socially, and participate fully in daily activities [14]. Empowered individuals are better equipped to advocate for their needs. They can actively seek appropriate interventions, communicate preferences, and make informed decisions about their hearing health. Empowered individuals learn effective coping strategies and adaptive techniques. They can navigate challenging situations, use assistive devices, and manage communication barriers more effectively [15]. Empowerment involves addressing emotional, psychological, and social aspects as well as hearing. Empowerment encourages a holistic approach to well-being and is a concept that is often raised in qualitative studies exploring the lived experience of adults with hearing loss [16].

To assess the impact of hearing rehabilitation on patient empowerment or determine whether an intervention has influenced a patient’s empowerment journey, it is essential to use a measure specifically designed for the relevant population. This measure should evaluate various dimensions of empowerment. Recognizing the increasing significance of empowerment in audiology research and the absence of a context-specific empowerment measure, Gotowiec et al. (2023) created a hearing-specific assessment tool for empowerment during the hearing health journey. The development of the Empowerment Audiology Questionnaires (EmpAQ-5 and EmpAQ-15) involved participatory methods, engaging both individuals with hearing loss and professionals working with them. The EmpAQ tap into the five empowerment dimensions: knowledge, skills and strategies, participation, control, and self-efficacy. There are two versions of EmpAQ: a fifteen-item version (EmpAQ-15) and a five-item version (a subset of EmpAQ-15; EmpAQ-5). The 15-item version is suitable for more in-depth research and/or clinical work, the five-item version is suitable for clinical applications given its brevity and for pre- post hearing rehabilitation interventions as it has no hearing aid-specific questions [17]. The items in the EmpAQ are constructed as statements with a 5-point Likert response scale (strongly disagree, disagree, agree, strongly agree) with an additional non applicable answer alternative placed at the right side of the scale [18]. The score is presented as a scale with a theoretical range of 0–100%, with higher scores indicating higher levels of empowerment. The rigorous process of designing and validating the EmpAQs adhered closely to evidence-based guidelines [19, 20]. Further, the EmpAQs have been tested through modern test theory (Rasch analysis) and Classical test theory (internal consistency reliability, construct validity and criterion validity), demonstrating acceptable results [17]. The English original versions of the EmpAQs are available for use at Open Science Framework, OSF (https://osf.io/caj84/).

The EmpAQs were developed in English, as most questionnaires used in audiology [21, 22]. However, there is clear applicability for clinical use of the survey in countries around the world, where clients speak languages other than English.

In recent years, Sweden has prioritized person-centered care across healthcare settings [23].Within the Swedish audiological clinical practices, there are various outcome measures used to various degrees. Most outcome measures are focusing on the use and benefit of hearing aids. However, currently there is no hearing related questionnaire addressing feeling of empowerment [24]. To facilitate the utilization of the EmpAQ within Swedish hearing health care services, it is imperative to establish a rigorously validated Swedish version of the questionnaire. Translations of assessment tools are essential for assessing a phenomenon across diverse linguistic and cultural contexts, as well as for making meaningful cross-country comparisons [25]. When translating questionnaire items, it is important to ensure that the translation accurately captures the intended concept, consistent with the original. Additionally, instructions and response options, except for the questionnaire items, should align with the experiences of the population of interest [26]. Ideally, a translated questionnaire should demonstrate functional equivalence, ensuring that its content aligns closely with that of the original questionnaire [27].

As for an original version of an instrument, it is recommended to evaluate the content validity of the translation and thereby address the instrument’s ability to adequately capture a construct [28]. According to the Consensus-Based Standards for the selection of health Measurement INstruments (COSMIN) guidelines for Patient Reported Outcome instruments, the content validity is evaluated by addressing relevance, comprehensiveness (if the content is covering relevant aspects of the measured construct), and comprehensibility (understanding and clarity) of an instrument [29, 30].

The primary objective of this study was to translate the EmpAQ from English to Swedish using best practice methodology. Additionally, the study aimed to assess the content validity of the Swedish version of the EmpAQ (EmpAQ 15-SWE) from the perspective of adults with hearing loss. As a secondary objective, the study evaluated the clarity of the instructions following the completion of EmpAQ-SWE as well as instructions for the result calculation to ensure that audiologists could utilize the questionnaire seamlessly.

## Materials and methods

The translation procedure and field testing of EmpAQ-15 SWE followed best-practice principles as described by Hall et al. (2018) where the translation procedure was divided into steps (i-iv) prior to field testing (v) and reviewing and finalizing the translated questionnaire.

First, all authors involved in the original questionnaire were contacted to secure their approval for the translation. The first author of this study (JL) was also engaged in the original English questionnaire development. Instructions to translators (appendix 1) and the interview guide for field testing (appendix 2) were created and ethical approval was obtained from the Swedish Ethical Review Authority (Dnr 2022-06176-01, 2023-01-11).

Secondly, two Swedish-English bilingual speakers were recruited for the forward translation.Both translators were native Swedish speakers, with one being a professional translator and the other a trained linguist and phonetician. They were provided with written instructions detailing the purpose and objectives of the translation process. Additionally, they received information about the concept of empowerment and an explanation of the empowerment dimensions as defined by Gotowiec et al. (2022). Their task was to ensure that the translated questionnaire items, instructions, response scales, and scoring guidelines from English to Swedish retained both the conceptual and semantic content, using everyday language. The independent translations were then combined by the authors (JL & EK), incorporating an audiological perspective.

Thirdly, an additional native Swedish speaking translator, with high proficiency in English, audiology, and linguistics, translated everything back to English. The forward and backward translations were compared, and discrepancies were assessed using an A-D scheme to prepare for the committee review and to highlight more complicated items. According to Hall et al. (2018), items demonstrating perfect semantic agreement were marked as “A,” while those without agreement were marked as “D”.

In the fourth and final step of the translation process, a committee was convened. A group of experts, including authors, a backward translator, and a licensed audiologist who had not previously participated in the project, aimed to create the Swedish version of the questionnaire (EmpAQ-SWE) that faithfully mirrored the original questionnaire in terms of content and meaning. They held two meetings, prior to and after data collection, where they reviewed translations, resolved discrepancies, and addressed issues identified during data analysis.

Detailed records were maintained through audio recordings and notes to document the discussions.

As a fifth step, the translated instrument underwent field testing. The objectives were to assess whether translated instructions, items, and scoring instructions were *equivalent* (i.e., showing correspondence in a conceptual, and semantical way) *accessible* (i.e., was clear and possible to understand), and *acceptable* (i.e., that the wording was perceived as acceptable and not offensive) compared to the original [21]. Also, the evaluation of content validity focused on assessing *relevance* i.e., targeting items found as irrelevant to the construct or to the target population, and *comprehensiveness* i.e., to identify if the target population found noticeable omissions as described by Polit & Beck (2017).

### Participants

For the field testing, ten participants (5 male, 5 female) were recruited from the research laboratory ORCA Europe’s participant pool. The selection process was conducted through purposive sampling, ensuring maximum variation based on age, gender, hearing-aid-usage time, and degree of hearing loss. The average age of the participants was 73 years, with a range from 53 to 90 years (SD 10.5). Since these participants were drawn from the participant pool, information regarding their hearing thresholds was already available, and they had previously given consent for the use of collected audiogram data during recruitment. The degree of hearing loss was classified according to the World Health Organization’s (WHO) criteria for hearing impairment, using the pure tone average for four frequencies (PTA4) at 500, 1000, 2000, and 4000 Hz in the better ear [9]. All participants were experienced binaural hearing aid users. Most participants used hearing aid applications to adjust the hearing aid sound. Additionally, all participants used assistive listening devices, which included external devices such as loop systems or TV solutions, or their mobile phones for streaming TV sound, phone calls, online meetings, audiobooks, and music. For a comprehensive overview of demographics, please refer to Table 1. All recruited participants were native Swedish speakers. None of the participants reported cognitive difficulties or challenges in reading and completing questionnaires, which were exclusion criteria for the study. Prior to data collection, participants received information about the study, and informed consent was obtained during the interview. No reimbursement was provided for their participation.

**Table 1:** Demographics for field testing participants

Demographics	Participants (N = 10)
Age, mean, (SD^1^; range)	73 (10.57; 53–90)
Gender, n (%)	
* Male*	5 (50%)
* Female*	5 (50%)
Hearing loss^2^, n (%)	
* Mild*	1 (10%)
* Moderate*	2 (20%)
* Moderately severe* * Severe*	5 (50%) 1 (10%)
* Profound*	1 (10%)
Years since *first* hearing aid fitting, mean, (SD; range)	20.9 (10.11; 7–44)
Years since *last* hearing aid fitting, mean, (SD; range)	2.7 (1.4; 1–5)
Use of ALDs^3^, n (%)	
* Yes*	10 (100%)
* No*	0 (0%)
Use of Hearing Aid APPs, n (%)	
* Yes*	8 (80%)
* No*	2 (20%)
Employment	
* Retired*	8 (80%)
* Working*	2 (20%)
Education years, n (%)	
≤ 9	1 (10%)
> 9 ≤ 12	1 (10%)
> 12	8 (80%)

^1^SD= standard deviation, ^2^WHO’s classification of hearing impairment, PTA4 best ear

Mild 20 to < 35 dB, Moderate 35 to < 50 dB, Moderately severe 50 to < 65 dB, Severe 65 to < 80, Profound 80 to < 95 dB. ^3^ALD= Assistive listening devices

### Data collection

Field testing data was collected by the first author (JL) through cognitive debriefing via one-on-one semi-structured interviews, utilizing the think-aloud technique [31]. The test leader was a licensed audiologist with previous experience in qualitative interviewing for research purposes. The interview process began by providing participants with information about the interview and introducing and practicing the think-aloud method. Participants were encouraged to articulate their thought process as they worked through the questionnaire, item by item. During data collection, the questionnaire items were presented on paper, maintaining the same layout as the original version. Participants were instructed to read the questionnaire instructions and paraphrase them in their own words. As for the questionnaire items, participants read them aloud, expressing their thoughts while marking their answers. The test leader introduced additional scripted and spontaneous probes after a set of items (grouped by empowerment dimensions) to gather insights on accessibility, acceptability, and relevance. The respondents were asked to choose from five alternatives: strongly disagree, disagree, agree, strongly agree, or not applicable when discussing each questionnaire item statement. At the end of the interview, participants shared their overall impressions of the questionnaire. They were specifically asked whether they thought that some aspect on empowerment was missing. This was later used as a measure of the overall comprehensiveness of the questionnaire. The first interview served as a pilot test for the interview guide and procedure. Since no changes were necessary, this interview was included in the dataset. All interviews were audio-recorded using two separate recorders. Transcriptions of the interviews captured participants’ responses verbatim, excluding non-verbal sounds like sighs and laughter. On average, the 10 semi-structured interviews lasted 44 min (with a range of 29 to 71 min).

As part of the field-testing process, audiologists with current or prior experience in hearing rehabilitation evaluated the instructions for completing and scoring the EmpAQ -SWE. Seven audiologists from various regions in Sweden, recruited through the authors’ professional network, participated in this evaluation. The audiologists were requested to assess how well they comprehended: the aim of the questionnaire, the definitions, the respondent task, and how to calculate the results. Additionally, they were assigned with calculating the score for a hypothetical respondent and engaging in a discussion about the results they obtained. Their feedback was communicated to the authors via email.

### Analysis

The ten individual transcripts from the participants’ interviews were treated as units of analysis according to Graneheim and Lundman (2004). Each transcript underwent a comprehensive review, involving both listening to the audio recordings and reading the transcribed text. This process ensured that the transcriptions corresponded to the interviews. Any transcription errors were corrected, and identifying information such as names of individuals or places was anonymized. Subsequently, the transcribed text files were uploaded into the qualitative data analysis software NVivo 13 by QSR International (2020) for further analysis.

Data underwent qualitative content analysis, focusing on manifest content combining both deductive and inductive coding approaches [32, 33]. Initially, the interview content was divided according to the different questionnaire items (1–15). The coding process involved dividing the text into meaningful units, which were then condensed into codes. These codes were subsequently clustered into sub-categories and categories. The deductive coding was supplemented with inductive coding to delve deeper into cultural nuances. Categories for each questionnaire item were analyzed and labeled with one overarching theme [33], see Table 2.

The equivalence was assessed by comparing each overarching theme with each questionnaire statement, evaluating the participants’ views in relation to the questionnaire statement. Through deductive coding, the researchers also explored how the test participants perceived accessibility i.e., clarity, and relevance for the different items as well as the general overall impression of accessibility, acceptability, relevance, and comprehensiveness of the entire questionnaire.

When analyzing accessibility, the participants’ responses were divided into three groups: clear and easy to understand, expressing some difficulty in answering the question, and difficult to understand. Also, data including information on questionnaire instructions and response scale was deductively sorted. To increase the trustworthiness, all data were coded and analyzed by the two authors separately from each other. Discrepancies in coding and interpretation were discussed and codes and themes were refined from the discussions.

## Results

The results from the translation process encompassing comparisons of the forward and backward translation and the committee review discussion are described below. Additionally, data which guided the exploring of content validity, i.e., equivalence, accessibility, and acceptability is presented. The content validity was also evaluated in terms of relevance and comprehensiveness. The questionnaire items related to the overarching themes are presented in Table 2.

### Translation process

In the comparison of forward and backward translations, guided by Hall et al. (2018), none of the fifteen questionnaire items were marked as ‘D’ (without agreement). Three items were classified as ‘A,’ indicating very similar forward and backward translations that easily reached consensus. Eight items were marked as ‘B,’ signifying satisfactory semantic equivalence but with one or two different words. An additional four items were labeled ‘C’ for preserving the meaning of the original but lacking satisfactory semantic equivalence [21]. Committee discussions primarily focused on the ‘B’ and ‘C’-marked items, particularly questionnaire items 1, 6, 7, and 8 (labeled ‘C’), as well as item 11 (labeled ‘B’). These discussions centered around semantic equivalence and language nuances. Additionally, questions arose about the original construction of items 3 and 9. To address these concerns, questionnaire item 11, along with items 3 and 9, were reviewed by native English speakers from the original developer group, guiding the final wording selection.

The committee thoroughly reviewed each translation item, including instructions, response scale, and scoring guidelines. Their primary objective was to reach consensus on a single Swedish translation. As a result of their discussions, the EmpAQ-SWE questionnaire was compiled.

### Equivalence

The assessment of equivalence involved comparing each overarching theme with every questionnaire statement. We evaluated participants’ perspectives in relation to these statements. Overall, these overarching themes closely corresponded to the dimensions of empowerment: knowledge, skills and strategies, participation, control, and self-efficacy.

For questionnaire item 1 to 4 (knowledge dimension), the participants discussed knowledge about how to manage communication problems, knowledge about how to get help, knowing where to find useful information (regarding hearing loss) and knowing where to find information (regarding hearing devices). For items 5 to 7 (skills and strategies) the participants discussed being aware of and using tactics, ways of coping with my hearing loss and applied hearing aid management. For items 8 to 9, (participation) the overarching themes were connections/relations to hearing health care and participation in relation to hearing loss. Items 10 and 12 (control) presented feeling in control and wanting to be in control over devices, and different aspects of control and effect of hearing devices. Following, for items 13 to 15 (self-efficacy) the overarching themes were level of confidence, level of confidence in relation to severity of problems, and being confident. Lastly, for questionnaire item 11 the overarching theme was “Focusing on control or challenges”, some but not all categories corresponding to the control dimension. For an overview of the questionnaire items, the generated overarching themes, and categories, see Table 2.

**Table 2:** Overview of the questionnaire items, overarching themes, and categories

Questionnaire items		Overarching themes	Categories
KNOWLEDGE			
1	I know how to manage communication problems caused by my hearing loss.	Knowledge about how to manage communication problems.	• measures for managing communication problems. • knowing how to manage communication problems is depending on the situation. • learning to manage communication problems is a process. • is not experiencing communication problems
2	I know how to get help if I have any problems with my hearing.	Knowledge on how to get help.	• knowing how to get help. • knowing how to get help varies from situation to situation
3	I know where to find useful information about hearing loss.	Knowledge about where to find useful information.	• being aware of places to find information. • being aware but not knowing if there are *other* places. • not being aware of places to find information
4	I know where to find useful information about my hearing device(s)	Knowledge about where to find information about hearing devices.	• finding information on hearing devices from the hearing health care • finding information on hearing devices from the hearing aid manufacturer • finding information on hearing devices from the internet
SKILLS AND STRATEGIES			
5	I use tactics to help me communicate in challenging situations (e.g., move to a quieter location).	Being aware of and using tactics.	• consciously using tactics • having tactics but refrain from using them in certain situations. • being impossible to use tactics,
6	I search for other ways to help me cope with my hearing loss when I need to (e.g., look online or ask a friend.	Ways of coping with hearing loss.	• coping by myself • turning outwards • have not been reflecting on ways of coping
7	I have the skills to clean, care, and manage my hearing device(s) and any accessories I may have.	Applied hearing aid management.	• cleaning hearing devices • maintaining hearing devices • acting preventively for hearing devices to work optimally
PARTICIPATION			
8	I contact my hearing care professional whenever I need anything.	Connections/relations to hearing health care.	• taking action for contact • not acting for contact • previous positive experiences of contacting the hearing health care
9	My hearing loss doesn’t stop me from taking part in social activities.	Participation in relation to hearing loss.	• hearing loss is not stopping me from taking part in social activities. • hearing loss is stopping them from taking part in social activities. • physical participation but with less involvement
CONTROL			
10	I feel in control of my hearing device(s) and accessories.	Feeling in control and wanting to be in control over devices.	• feeling in control over how the hearing aids are working technically. • feeling in control regarding how to manage my [i.e., their] hearing aids. • wanting to be in control of one’s hearing aids and accessories
11	I can control how I respond to the challenges I experience resulting from my hearing loss	Focusing on control or challenges	• being in control over their responses to challenges • talking about challenges.
12	My hearing device(s) help me gain control over my hearing difficulties.	Different aspects of control, and effect of hearing devices	• using hearing devices is increasing feeling of control. • degree of hearing loss is affecting the feeling of control. • not feeling in control • hearing devices are relieving hearing difficulties
SELF-EFFICACY			
13	I am usually confident asking people to change how they talk to me when I need to (e.g., loudness)	Levels of confidence	• feeling [confident] or being confident • not feeling confident • feeling of confidence varies depending on the situation.
14	I am confident about my ability to manage problems caused by my hearing loss”	Level of confidence is related to degree of hearing loss and situation.	• feeling confident • not feeling confident • depending on the situation, feeling more or less confident.
15	I am confident telling my hearing care professional what is important to me.	Being confident in contact with hearing care.	• being confident

### Knowledge

#### Knowledge about how to manage communication problems

Within this theme participants discussed **measures for managing communication problems** describing managing communication problems by using different strategies. Some participants described functional strategies as informing others about what facilitates communication, not to turn away when talking or informing others that they need to be in the same room when talking. Other participants were telling about malfunctional strategies as pretending to hear, letting someone else handle phone calls, or just stop listening to others.

*And if there are a lot of people talking - then I don’t listen. I can’t keep up* (Female, moderately severe hearing loss).

Some participants also described withdrawing from situations which they perceived as difficult. When being impossible to influence a situation, they described that they managed by accepting the situation. Some participants were also talking of managing communication problems technically, describing usage of hearing aids and changing of settings in hearing aids.

There were participants explaining that **knowing how to manage communication problems is depending on the situation**. Some participants described that the ability to manage communication problems is dependent both on the specific situation as well as from the behavior from others.

*and of course there are situations where you have communication problems……so I had an intern at our job……and he knows that I have hearing aids*,* but he might not understand how bad my hearing is*,* if you say so*,* or how important it is to speak clearly.* (Male, moderately severe hearing loss).

Some participants who had experienced hearing loss for an extended period perceived that ***learning to manage communication problems is a process*** which becomes easier with time, age, and experience. One participant said that she in theory knows what to do but that she ***is not experiencing communication problems*** currently. Consequentially, this kind of knowledge is not useful right now.

#### Knowledge on how to get help

Within this theme some participants clearly confirmed ***knowing how to get help***, often by contacting the health care services i.e., their audiologists or the hearing health care clinic where they already have an established contact. Other given examples were using the internet to find a close-by hearing health care clinic or contacting the family doctor for a referral to an audiologist. However, some participants expressed that ***knowing how to get help varies from situation to situation*** and sometimes it is difficult to know whom to turn to.

*I sometimes think about when I stand on the commuter train*,* and they shout something into the microphone. You can hear them talking but … And there I don’t know who to ask*,* like. What do they say?* (Female, moderately severe hearing loss).

#### Knowledge about where to find useful information

Regarding this theme some participants described that they are ***being aware of places to find information***. Among the suggestions for where to find information about hearing loss is through the hearing health care services, in newspaper articles, on the internet but also through the hearing loss associations. Others express that they are ***being aware but not knowing if there are other places*** to find information.

*there might be someone else … some other place that has information that I don’t know about … … but then there might be some hidden things that I don’t know about.* (Female, moderately severe hearing loss).

Some participants said that they were ***not being aware of places to find information***. Additionally, some participants had not even searched for information on hearing.

#### Knowledge about where to find information about hearing devices

In relation to this theme, similar as for reporting on finding information about hearing, the participants described ***finding information on hearing devices from the hearing health care*** which could encompass both the hearing health care clinic and their personal audiologist.

Participants also reported ***finding information on hearing devices from the hearing aid manufacturer*** i.e., from the hearing aid app or in the hearing aid manual.

*I received a manual with the deliveries of the new devices*,* so of course I also looked at that. So*,* for the actual use*,* I have probably relied mostly on the manual that I received.* (Male, moderately severe hearing loss).

Some participants also described ***finding information on hearing devices from the internet*** googling for information.

### Skills and strategies

#### Being aware of and using tactics

Within this theme participants described how they are ***consciously using tactics***. A range of examples were shared as moving to a quieter location, moving closer to the sound source or to the person they want to communicate with, and, assuring that the communication conditions are as optimal as possible for both vision and hearing. Verbalizing the difficulties for example asking to turn off music or telling people to postpone talking until reaching quieter surroundings were also exemplified.

One participant shared that his family is aware of his hearing loss and are facilitating the situation for him by for example being clear on where they want to be placed when making table reservations. Sometimes participants described consciously using tactics that where they redraw from the situation by for example answering “yes” when not hearing or just listening, refraining from talking when being in a group, just not to make a fool out of themselves.

*…it’s difficult when there are many people talking*,* to hear*,* so you have to move a bit closer or simply choose not to hear*,* and then you can respond with ‘yes’.* (Female, mild hearing loss).

Some participants reflected on how they sometimes are ***having tactics but refrain from using them in certain situations***, for managing the situation there and then by, for example only talking to the closest-by person or the person having the easiest voice to hear. Or answering “yes” when perceiving that the conversation as less important. Some participants also revealed that sometimes it is ***being impossible to use tactics***, depending on the situation.

*But it also depends on the fact that in that case*,* you can say … … if you are among a group at a café*,* for example. Then I can’t say*,* shall we go and sit somewhere else.* (Female, moderately severe hearing loss).

#### Ways of coping with hearing loss

In relation to this theme participants talked about ***coping by myself***, commenting that they seldom would ask a friend for advice or saying that friends (as in the item example) do not know about their hearing loss. Additionally, they expressed that a way of coping by themselves could be shifting sensory modalities i.e., using other senses than hearing to a higher degree for example reading more instead of listening.

*You sort of increase the consumption of certain media*,* and decrease*,* then I cut back on other things. That’s the handling you do*,* huh.* (Male, moderately severe hearing loss).

Others talked about ***turning outwards***, i.e., turning to others to see if they have heard what’s being said, contacting the hearing health care, or using internet more for information. Some participants expressed that they ***have not been reflecting on ways of coping*** adding that the questionnaire item made the participant reflect on other ways to cope with hearing loss.

#### Applied hearing aid management

In this theme participants talked about ***cleaning hearing devices*** knowing how to clean their devices as well as their routines for doing so. Participants also described ***maintaining hearing devices*** as changing wax filters, tubes, filters, and recharging the devices. For this questionnaire item the participants also discussed ***acting preventively for hearing devices to work optimally*** where they for example described having an extra pair of hearing aids at home for safety’s sake, remembering to bring chargers when travelling, or going on regular technical checkups to assure that the hearing aids are working as they should.

*Once every six months*,* I usually contact a hearing center and get to go there to test that the hearing aids are working.* (Male, moderate hearing loss).

### Participation

#### Connections/relations to hearing health care

For this theme participants were talking about ***taking action for contact***, where many participants described the hearing health care as a go-to-place when needing any assistance. Others also talked about contacting the hearing aid manufacturer directly for technical assistance or bringing up the need for contacting different people for different problems, i.e., purely technical, or medical. Some participants said that they were ***not acting for contact*** for various reasons, i.e., or for not knowing if there would be other places to get help with more general hearing related questions, not being hearing aid specific or being hesitant contacting the hearing health care, mainly for it being difficult to get an appointment or receiving help with purely technical issues.

*Then in general*,* I think that if you have any kind of problem with your hearing aids and need help*,* and I unfortunately have to say that my supplier is a bit difficult because they have such a busy schedule*,* so I have been reluctant to contact them* (Male, moderately severe hearing loss).

Additionally, one participant talked about ***previous positive experiences of contacting the hearing health care***, where he before had visited a center for more general hearing related questions which he now lacked in his current hometown.

#### Participation in relation to hearing loss

Within this theme some participants confirmed that ***hearing loss is not stopping me [i.e.***,*** them] from taking part in social activities***. They mentioned that hearing aids or other accessories allow them to hear well enough to for example going to bigger events or to the theatre. Other participants said that ***hearing loss is stopping them from taking part in social activities***, describing how they were refraining from lectures, concerts and theatres or refraining from taking part in associations, or conversations. Some described feeling socially isolated in certain situations or resigning from certain tasks as for example being board members in associations.

*I am part of the condominium association here where we live and I’m a member of the board*,* and then we almost always have our meetings via Zoom and so online……and I’m thinking of dropping out because it doesn’t really work when so you can’t hear perfectly.* (Male, moderately severe hearing loss).

Some participants also experienced feeling ***physical participation but with less involvement*** i.e., still going to social activities but sometimes being less engaged.

*Then I take part in social activities*,* but I don’t always gain no benefit from it* (Male, profound hearing loss).

### Control

#### Feeling in control and wanting to be in control over devices

Regarding this theme the participants were discussing in rather practical terms as ***feeling in control over how the hearing aids are working technically*** i.e., by knowing and understanding hearing aid functionality, knowing how to adjust the sound in the hearing aids or knowing how apps and streaming works.

*the app*,* actually*,* you could say it’s an assistive device. And I think that*,* just that you have control of that you can get the sound directly into the hearing aid*,* that’s tremendously good*,* actually. And I know what to do when it doesn’t work*,* so I have control over that too.* (Female, moderately severe hearing loss)

Participants also expressed ***feeling in control regarding how to manage my [i.e.***,*** their] hearing aids***, i.e., placing them on the ears, cleaning and changing spare parts. There were participants clearly expressing ***wanting to be in control of one’s hearing aids and accessories***. Except for expressing the wish for adjusting hearing aids themselves to a higher degree to feel more in control, they also expressed feelings of losing control in relation to not being able to adjust their hearing aids so they can hear, or when for example hearing aid app functionality is poor.

*But the flaws in the app could be interpreted as causing me to lose a bit of control. For example*,* when I’m talking on the phone*,* I can’t change the volume of the devices via the app*,* and I think that’s a shortcoming*,* and it makes me not have complete control.* (Male, moderately severe hearing loss).

#### Focusing on control or challenges

This overarching theme gave a more diversified discussion than for other items. Some participants expressed that they felt as ***being in control over their responses to challenges***. One participant was explaining that being aware of the disability (having a hearing loss) was enabling for having increased control over the current situation. Another participant was explicitly confirming having control over emotional challenges i.e., control over her feelings in relation to her hearing loss.

*…that I manage by not being sad*,* hurt*,* that fits very well. If that’s what you’re asking for? So*,* I can control that I don’t get angry or start crying. But I can also be sad inside. I can also be - I don’t care about it. So yes*,* at large*,* I can control my emotions.* (Female, moderately severe hearing loss).

Participants also talked about acceptance, explaining that after doing what is possible to improve the situation, one response to the challenge is letting it pass or accepting the situation. Other participants were ***talking about challenges***, instead of how they felt being in control over their responses to challenges. They were reflecting on what challenges are, giving examples as not being able to hear, the sound being too loud, or the behavior or reactions from the others, i.e., when someone showing irritation when the participant cannot hear.

*…challenges can also be when someone gets annoyed when you can’t hear. It happens*,* often when you have said “what?” two or three times. Then they scream instead*,* or something like that.* (Female, moderately severe hearing loss).

The participants also described reactions to the actual challenges, for example being sad but not showing it, pretending to hear, feeling stupid, being socially isolated. When talking about challenges, the participants discussed the inability to control difficult situations and the difficulties in control the challenges sometimes. Some expressed that they do not think it is possible to be in control. Another participant expressed that controlling becomes more difficult with age as the hearing loss worsens.

*I think it’s very difficult to control how*,* how … in a lot of different situations where you*,* where you depend on your hearing … … Yes*,* but it might be a little easier if you’re a little younger and your hearing is not as bad as my hearing is. Because the older you get*,* the worse it gets … it’s been decreasing all the time and it’s difficult.* (Female, moderately severe hearing loss).

Also, the participants described how they control challenges rather than how they control how they respond to challenges, saying that they control challenges by for example placing oneself where it is possible to hear, avoiding situations or using tactics.

#### Different aspects of control, and effect of hearing devices

For this theme some participants agreed that ***using hearing devices is increasing feeling of control*** over hearing difficulties. The participants gave examples as being able to communicate with significant others, having social contacts, keeping up with what is happening in the world, and opening up the world.

*Yes*,* it’s the best hearing aid I’ve got……So it’s a new world that opens up. So you should … you should get hearing aids*,* it’s important*,* for being involved*,* because today everything happens so fast*,* if you don’t keep up with the development or what’s being said*,* then you’re left out.* (Male, moderate hearing loss).

That the ***degree of hearing loss is affecting the feeling of control*** was raised where one participant expressed that gradually loosing hearing made him gradually losing control.

*…then it was with a milder hearing loss. Then*,* in general*,* you had kind of better control over the environment and everything*,* and the hearing aids don’t really have that much importance then*,* it works well anyhow. But the more you become dependent on the hearing aids*,* the worse I think it actually gets.* (Male, moderately severe hearing loss).

Some participants expressed that they were ***not feeling in control*** questioning if it is ever possible to be in control, others did not know how to get control over their hearing difficulties or being more neutral expressing not feeling in control but in some ways accepting that.

*It’s just stating that my hearing is bad and I’m doing what I can to make everything better. So that*,* I think*,* it is difficult to get that control.* (Female, moderately severe hearing loss).

Some participants were talking about hearing rather than control, expressing that ***hearing devices are relieving hearing difficulties***. Participants expressed examples as that hearing aids are helping with hearing difficulties, helping them hear what other people are saying where not hearing was like being in a vacuum.

### Self-efficacy

#### Levels of confidence

Regarding this theme some participants shared that they are ***feeling [confident] or being confident*** asking people to change how they talk, often explaining to others that they cannot hear, asking someone to adjust their position so that it is possible to lip-read. Others were emphasizing that they are not feeling embarrassed over their hearing loss. Additionally, some participants described being a process towards becoming more confident with increasing age and experience. Also expressing it being easier after retirement when hearing everything is less important than in a working situation.

Other participants expressed that they are ***not feeling confident*** to ask people to change the way they talk, giving examples as perceiving that others could think of them as being stupid for not hearing or that people get irritated when being asked to change how they talk.

Some participants said that the ***feeling of confidence varies depending on the situation*** where it could be difficult asking unknown people to change how they talk, or being less confident and feeling that it is more difficult to ask in a situation where many people communicate.

*But if you sit in a group like when I was working*,* there were maybe ten people and there was a discussion about something. And then you can’t say: well*,* hey*,* let’s take it one more time*,* because then you become a cumbersome person*,* so then you bite the bullet and then you try to keep up with the times*,* anyway* (Male, moderate hearing loss).

#### Level of confidence is related to degree of hearing loss and situation

Within this theme when discussing the item “I am confident about my ability to manage problems caused by my hearing loss”, some participants confirmed that they are ***feeling confident*** about their ability. When reflecting on why, examples as being successfully fitted with hearing aids and therefore having sufficient help to hear or not experiencing big difficulties, came up. There were also participants revealing that feeling confident about their ability is an ongoing process that has become easier with increasing age. One participant said that living with hearing loss for some time has brought a certain ability to handle problems.

On the contrary, there were participants who expressed ***not feeling confident*** at all, for example in relation to situations which were in the hands of others, some talked of trying to accept losing a bit of the world. One participant talked about being afraid of discovering that something they said was misinterpreted.

*I wonder if someone who is hard of hearing can feel confident about it [their ability to handle problems]……No*,* because if you feel confident about that*,* then maybe your hearing is not so bad. I think. My hearing is so bad that I find it difficult to handle.* (Female, moderately severe hearing loss).

Some participants said that they are ***depending on the situation***,*** feeling more or less confident*** and feel more insecure with unknown people or when they are not able to influence a specific situation.

Furthermore, some participants also were emphasizing that it is not always possible to handle problems when the problems they are experiencing is connected to the behavior of others or when the severity of the hearing loss is too big.

*No*,* because if you feel safe with it*,* then you might not hear so poorly. I think. My hearing is so bad that I find it difficult to handle.* (Female, moderately severe hearing loss).

#### Being confident in contact with hearing care

In this theme participants shared that they are ***being confident***. One participant shared that she had good contact with the hearing care professionals and therefore were being able to talk with them about important topics. Participants also established that telling what you want is a prerequisite for getting the help that you need. Some participants concluded that as the hearing care professional are there for the patients, there is no reason not telling them what is important. Some participants described getting increased confidence with time and age expressing that is has been a learning curve and that confidence have come with increasing knowledge and age.

*I have to do that to get the right help.…it might have to do with confidence. But that’s sort of why you go to the staff*,* to get help.* (Female, mild hearing loss).

### Accessibility

Questionnaire accessibility was assessed by asking the participants to elaborate on their views of the clarity of each item. From their responses the questionnaire items were divided into groups, see Table 3.

**Table 3:** Accessibility of questionnaire items

Participants view on accessibility	Questionnaire items
The question is clear and easy to understand	2, 3, 4, 5, 7, 8, 13, 15
Expressing some difficulty in answering the question	1, 6, 9, 14
Difficult to understand	10, 11, 12

A majority of the questionnaire items were thought to be clear and comprehendible. Items belonging to the empowerment dimensions knowledge, skills and strategies, participation, and self-efficacy, were mostly perceived as clear and easy to understand by the participants. In most cases, when the participants expressed that the items were clear and easy to understand they did not expand their reasoning further by giving examples. For some the questionnaire items some participants expressed difficulties in answering the items, after being asked about clarity of the questions. Either due to their interpretation of the question in relation to their situation where for item one, one participant said:

*I actually do not really know how to … if one should handle communication problems in different ways. I mean*,* what can I do? If I come into a place where there is a loop*,* then I can press so that I can use the loop amplification*,* eh. But in many situations*,* there is simply … then you can’t handle it*,* simply put. There’s nothing to be done about it*,* it simply doesn’t work.* (Male, severe hearing loss)

or as for item 6 one participant reacted to how the item was constructed:

*What does ‘coping with my hearing loss’ mean? I mean*,* if you google it on the internet*,* I won’t hear better because of that. Yes*,* I have to think a bit. The answer is ‘not at all’ because I don’t see any way to coping with my hearing loss when needed. But still*,* I think it’s a strange question.* (Male, moderately severe hearing loss).

For questionnaire item 9 “my hearing loss doesn’t stop me from taking part in social activities” one participant marked the answer differently than how the participant was speaking about the questionnaire item. Other participants recognized this difficulty and one suggested removing the “not” in “doesn’t stop me” reasoning that double negations could be difficult to interpret in items. Items 10, 11, and 12 explore the empowerment dimension control. Although clear for some participants, others expressed difficulties in interpretation. Specifically, for item 10 “I feel in control of my hearing devices”, one participant questioned whether the item aimed to investigate control over the physical location of the devices or ownership.

*That sounds really strange. That I have control over … is it that I have control over that they are my own*,* or that I have control over where they are. That I have control over how they work. No*,* that was a difficult*,* strange question.* (Female, moderately severe hearing loss).

Additionally, some participants noted that the questionnaire item appeared to overlap with previous questions related to skills and strategies. For questionnaire item 11 “I can control how I respond to the challenges I experience resulting from my hearing loss” one participant found the question challenging due to its inclusion of multiple concepts.

*It is difficult*,* that question. Because the question actually consists of several different things. Control*,* handle*,* challenges I experience. So*,* you really get … it’s too much at once*,* you might say*,* in that item.* (Male, moderately severe hearing loss).

### Acceptability, relevance, and comprehensiveness

Overall, participants found the language of all questionnaire items to be acceptable. However, when it came to self-efficacy, items 13, 14, and 15 evoked negative emotions for two participants. They expressed difficulty in discussing self-efficacy in the context of their hearing loss, leading to feelings of sadness. All participants but one confirmed all items as being relevant. This participant perceived items (10 to 14 control and self-efficacy dimensions) as overlapping to questionnaire item 5 (strategy dimension) and thus less relevant to the participant. The participants mostly described the questionnaire as comprehensive. However, a few potential improvement suggestions came up. One participant noted the absence of the perspective of significant others and how this affects the feeling of empowerment. Another participant expressed a wish for long-term support during the hearing journey to be addressed. A third participant mentioned the contextual aspects of many items and wished for these to be included somehow.

### Instructions, scale, and scoring

During the discussion about the questionnaire instructions, participants reflected on both the definitions provided and the guidance on how to complete the questionnaire. When discussing the concept of empowerment, some participants expressed that it was a novel concept for them. In EmpAQ-SWE, the word hearing care professional was translated to hearing care (hörselvård), as it is more usual that people with hearing loss visit a place (hearing care clinic) than a specific audiologist. One participant reacted to the word saying that they more perceived getting hearing aids than taking part in hearing care.

During the interviews, participants expressed varying opinions about the response scale used in the questionnaire. Some participants found the scale to be acceptable and comparable to other questionnaires they had encountered before. Other participants felt that a mid-alternative between “disagree” and “agree” was missing. In general, the audiologists indicated that they comprehended the instructions and definitions related to empowerment, hearing aids, and hearing care. All audiologists reported on how to calculate and interpret the EmpAQ-SWE scores in a correct way, although one audiologist ended at the wrong score which could imply the need for an automated scoring when distributing the EmpAQ-SWE in a clinical setting.

## Discussion

This study aimed at translating and culturally adapt the Empowerment Audiology Questionnaire (EmpAQ-15) from English to Swedish and to investigate the content validity of the Swedish version from the perspective of adults with hearing loss. Content validity was assessed by examining equivalence, accessibility, acceptability, relevance, and comprehensiveness.

### Equivalence

The statement ‘I can control how I respond to the challenges resulting from my hearing loss’ sparked more diverse discussions compared to the other items. Participants engaged in conversations about different aspects of this question. As one participant pointed out, the item contains much information to relate to i.e., *control*,* respond*,* challenges*. This could be a reason for the divided attention shown in the analysis. This divided attention could influence a measure’s test-retest reliability as well as interrater reliability [28] which has not been investigated within the scope of this study. Comparing the Swedish translation to the English original, both items are built up in the same way. It is therefore possible that the translation and original item will be affected in the same way. In upcoming studies, it is desirable to conduct additional test-retest reliability testing in both languages. This will help ensure the consistency and stability of the assessment tools used for measuring language-related constructs.

### Accessibility

Knowledge, skills and strategies, and participation are topics commonly discussed topics in a hearing rehabilitation context. As most items within these dimensions were perceived as clear and easy to understand by all participants, this could imply that more tangible topics are easier to understand and discuss than a more abstract construct as control. Previous studies regarding rehabilitation settings have shown that topics such as psychological and personality issues are more difficult for patients to relate to, compared to skills and strategies [34, 35]. However, the group of experienced hearing aid users were also able to relate to self-efficacy in relation to their hearing loss, especially when the connection to hearing rehabilitation was explained, discussing the items about confidence in relation to the contact with hearing care professionals. For some items, some participants said that they understood the question but found it difficult to answer. More information is needed to determine whether this difficulty is related to the word choice in the Swedish translation or to the participants’ personal experiences living with their hearing loss. Participants found the questionnaire items related to the control dimension (items 10, 11, and 12) to be the most challenging to understand. This dimension deals with an abstract concept, which contrasts with more concrete concepts discussed in the same context. Previous research also supports this observation, emphasizing that control is a complex topic for discussion [8]. Additionally, in Swedish clinical audiological settings, discussions about control are relatively uncommon, making it less familiar for participants to consider [36].

For item 9 “my hearing loss doesn’t stop me from taking part in social activities”, one participant marked the answer differently than how the participant was speaking about the item, i.e., answering the statement as it would have said “my hearing loss stops me”. Four additional participants recognized this difficulty and changed their marking while discussing. The remaining five participants showed immediate alignment between how they discussed the questionnaire item and how they marked it. This suggests that it could be problematic to include a negative barreled item in the questionnaire. Questionnaire construction literature often suggest not including double negative questions they could be confusing for respondents. These type of questions can take longer time to process, hence, increasing likelihood of responding differently than intended [37]. This risk was also discussed in Bennett et al. (2023) for the original item construction. However, Bennett et al. (2023) argued that data from the content evaluation showed preference for both versions and the decision to go with the negative barreled question was to accommodate for not having to reverse the scale when scoring the items. Guided by the insights from the Rasch analysis, the decision was made to retain the double negative construction while examining data from a large sample of people [17].

It is also worth considering that the cognitive interview method itself may pose a barrier for certain types of items. During a cognitive interview, participants have time to think, reflect, and even discuss their thoughts related to an item. During reasoning and discussion, respondents might deviate slightly from the exact wording of the item statement. In contrast, when participants complete a survey in a real-world setting, people usually read and answer a question more promptly.

### Relevance and comprehensiveness

Most participants confirmed the relevance and comprehension of all questionnaire items, suggesting that the content evaluation of the translated questionnaire is sufficient. Some participants perceived certain items as overlapping. For instance, for one participant, items 10 to 14 (related to control and self-efficacy) were seen as overlapping with item 5 (about skills and strategies). Interestingly, this participant had a mild hearing loss, also scoring on the higher end on the EmpAQ-SWE, which might indicate that issues around control and self-efficacy was less applicable to the participant.

For questionnaire item 1 “I know how to manage communication problems caused by my hearing loss” some participants answered that they knew what to do and managed by using tactics. This was also referred to for item 5 “I use tactics to help me communicate in challenging situations (e.g., move to a quieter location)”. That participants mention tactics in response to both of these items might indicate an overlap between them, potentially impacting the face validity of the instrument [38]. However, using tactics is relevant in connection to having a hearing loss [39] so it is not surprising that participants are referring to strategies both when being asked about tactics (item 5) and when being asked about if they know what to do to manage their communication problems (item 1). Also, when talking about the control dimension, some participants were discussing how they control challenges (by using tactics) rather than talking about how they control how they respond to challenges emotionally. From the current study it is not possible to explain why some participants circled back to certain ways of dealing with their hearing loss e.g., by using strategies when talking about different empowerment dimensions. Possible reasons could be that strategies are often referred to among ways of coping with hearing loss and that control is more seldom addressed during the hearing rehabilitation. However, depending on the participants degree of difficulties, it is possible solving hearing related challenges by using strategies whereas for others, other dimensions as control or self-efficacy is more relatable. Further studies on, for example, impact of degree of hearing loss are needed to understand this relationship, which is of relevance for both the English and Swedish version of EmpAQ.

When talking about tactics, participants discussed the different strategies they are using. These were in the analysis divided into adaptive and maladaptive, a terminology also used by Gomez and Madey [40]. Gomez (2001) describe adaptive strategies as behaviors that improve communication (i.e., asking others to repeat). The maladaptive strategies are described as coping behaviors that do not promote communication (i.e., pretending to understand the conversation). Similarly, in the study of Gotowiec et al. (2022) when conceptualizing empowerment for people with hearing loss, different strategies, adaptive and maladaptive were identified. The EmpAQ questionnaires are asking about respondents’ use of strategies but do not separate different types of strategies. This needs to be captured in other ways and could be an argument for using EmpAQ as a communication tool or as an outcome measure in combination to talking to the patient about which type of strategies they use.

In the translation process, the connotation of the word “challenges” was discussed, with alternative suggestions including “difficulties” or “problems”. These words, however, would probably be perceived more negatively and stigmatizing, not suitable in an empowering context. The word challenges in Swedish (*utmaningar)* is difficult as it could open up for different interpretations. Also seen in the data, some interpreting “challenges” being their hearing difficulties whereas others were more referring to problematic situations or environmental factors being challenging. Opening for interpretations could negatively affect the reliability of the instrument as the respondents may answer the items differently at different times [38]. On the other hand, it could be seen as a strength that many people, regardless of where in their hearing journey they are, can relate to an item and rate it from what it means to them.

The word *audiologist* was translated to the Swedish word for *hearing care* (*hörselvård)* and defined as businesses that provides hearing diagnostics and rehabilitation, where audiologists are working. This translation could be seen as a cultural adaptation since it in Sweden is common that you visit an office where you meet an audiologist and/or other professions as technicians, pedagogues, and psychologists. However, the organizational setups are different in different cities or between private or publicly founded business [41]. It is possible that different organizational setups might affect both how a patient look at their rehabilitation, i.e., as being customers buying hearing aids or receiving hearing loss rehabilitation (treatment) as well as how the EmpAQ should be administered. Using EmpAQ as an in-depth clinical tool and as basis for discussion might be more appropriate in audiological settings where a clinician provides holistic support and can provide information and support for the aspects of empowerment identified by the questionnaire as lacking. Addressing comprehensiveness, instead of altering the translated questionnaire to attend to the suggestions for incorporating other perspectives, these valuable comments could be utilized for example to develop and use the questionnaire as a discussion tool.

### Strengths and limitations

The participants, recruited from a participant pool, may not fully represent all individuals with hearing loss. Their experience in participating in studies and answering surveys exceeds that of the general population. Additionally, participants from participant pools may have received training in critically evaluating problems or questions. During the original development of the EmpAQ, participants were also recruited from participant pools in the United States and Australia [17, 42]. Despite potential cultural differences, the Swedish participant pool likely mirrors the population for which the EmpAQ was designed.

The recruitment process involved purposive sampling, considering age, gender, hearing-aid-usage time, and degree of hearing loss. While there was variation in gender, age, and hearing loss, the sample skewed toward retired individuals (80%) and those with post-high school education (80%) which could affect the level of comprehension of the questionnaire. The sample included experienced hearing aid users with moderate to severe/profound hearing losses but lacked newly fitted first-time hearing aid users. While our data provided rich insights and discussions on living with hearing loss and the gradual changes in hearing over a lifetime, aspects related to individuals who are new to hearing loss and their adjustment process in relation to empowerment is missing in our data.

When using cognitive debriefing with semi-structured interviews for questionnaires, it is essential to consider the limitations inherent to the method. Participants, while thinking aloud and reasoning during their responses, may inadvertently prolong or overthink their answers. The process introduces a time delay between reading the questionnaire item and providing a response, potentially impacting the scoring. While scoring data can offer valuable insights into participants’ reasoning, caution is necessary, particularly for items that prove challenging for participants to comprehend.

## Conclusion and future directions

The results demonstrated that most questionnaire items showed semantic and conceptual equivalence and were perceived as accessible, acceptable, and relevant. One item sparked discussion, as the elicited responses from the participants indicated a split focus on different aspects of the item. However, this could not be derived to the translation of the item. Instructions and scoring scales were at large perceived as relevant and comprehensive by audiologists. The comprehensive Swedish versions EmpAQ-15 SWE and EmpAQ-5 SWE, complete with instructions and scoring details, are openly accessible on OSF (https://osf.io/x79v5).

The current study investigating content validity of the EmpAQ-SWE is an important step preparing for further studies on validity and reliability in a larger sample of the population. In later stages, the questionnaires should preferably be tested in different populations, e.g., new hearing aid users, people with different degrees of hearing loss, comparing data collected in Swedish with data from English speaking populations.

## Electronic supplementary material

Below is the link to the electronic supplementary material.

Supplementary Material 1

Supplementary Material 2

## Data Availability

Swedish versions of Empowerment Audiology Questionnaire EmpAQ SWE are found openly available at OSF https://osf.io/x79v5/.
