# Supplementary material for: Swedish translation and content evaluation of the Empowerment Audiology Questionnaire (EmpAQ—15)
Source: J Patient Rep Outcomes. 2024 Dec 17;8:143. doi: 10.1186/s41687-024-00819-4 (PMC11652430; doi:10.1186/s41687-024-00819-4)
Supplement: Supplementary file 1 — Supplementary Material 1 [file 41687_2024_819_MOESM1_ESM.pdf]

Information for the Swedish Translation of the Empowerment Audiology Questionnaire (EmpAQ)

In recent years, interest has grown in research and health policy efforts related to patients' experience of "empowerment." While "empowerment" could be translated to "egenmakt" in Swedish, there is no widely accepted translation for the term. The concept of "egenmakt" refers to an individual feeling that they have control over personal, socioeconomic, and environmental factors that impact health (Elgán, 2014). The context determines how one discusses the concept, but the common thread is that empowerment enables an individual to feel mastery over a situation that affects them (Zimmerman, 1995).

Zimmerman (1995) suggests that there are several components, each of which is necessary for experiencing empowerment. An individual must perceive that they can influence a given situation (intrapersonal component), possess skills and knowledge to understand the situation (interactional component), and then behave in a way that affects a specific outcome (behavioral component). Within these components, there are dimensions relevant to experiencing empowerment from a psychological perspective: knowledge, participation, skill, control, and self-efficacy (Zimmerman, 1995).

Previous research on empowerment has primarily focused on chronic health conditions such as diabetes and cancer, and studies have shown a correlation between a high sense of empowerment and positive health outcomes (McAllister, Dunn, Payne, Davies, & Todd, 2012; Wallerstein, 2006). Since hearing loss can typically be classified as a chronic health condition, it is interesting and important to study empowerment in relation to hearing and hearing rehabilitation. However, the concept of empowerment is relatively new within hearing research. A literature search conducted by Gotowiec et al. (2022) in connection with their study on empowerment and hearing revealed that there were few published articles investigating empowerment in relation to hearing (Gotowiec et al., 2022).

In Gotowiec et al.'s (2022) study, the experience of empowerment was examined among individuals who had recently undergone hearing rehabilitation. The authors were interested in how participants perceived empowerment—from the initial awareness of their hearing loss, throughout the process of hearing rehabilitation, and as they became accustomed to using hearing aids. The study aimed, among other things, to conceptualize empowerment within the context of hearing.

In the study by Gotowiec et al. (2022), the experience of empowerment was examined among individuals who had recently undergone hearing rehabilitation. The authors were interested in how participants perceived empowerment—from the initial awareness of their hearing loss, throughout the process of hearing rehabilitation, and as they became accustomed to using hearing aids. The study aimed, among other things, to conceptualize empowerment within the context of hearing.

Based on data from semi-structured interviews of 18 people who had used hearing aids between 6-36 months, the authors conceptualized empowerment as: "a process by which individuals with hearing-related challenges acquire and use knowledge, skills and strategies and

APPENDIX 1: Note that the blue text in this document was originally presented in Swedish. An online translation was performed solely to ensure accessibility for reviewers and readers.

increase self-efficacy, participation and feeling of control over their hearing care, their hearing solutions and their everyday life". Based on Zimmerman's framework, the dimensions relevant to empowerment during the auditory journey are: knowledge, abilities and strategies, participation, control, and self-efficacy (Gotowiec et al., 2022).

As a natural consequence of understanding how empowerment is expressed within a hearing context, Gotowiec et al. also find a way to measure empowerment. A subsequent project therefore developed a questionnaire for empowerment, the Empowerment Audiology Questionnaire, EmpAQ (Gotowiec et al., under review; Bennett et al., 2022 in prep).

In Gotowiec et al, 2022, the different dimensions of empowerment are described as follows:

<u>Knowledge</u>
Knowledge refers to the acquisition and assimilation of information, leading to an understanding of an individual's hearing, hearing related challenges and hearing solutions.

<u>Skills and strategies</u>
Individuals use skills and strategies together to manage their hearing and improve communication with others.

<u>Participation</u>
Participation is the active involvement in both hearing rehabilitation and all aspects of social life, including family and informal social relationships. It encompasses the decisions, processes, and actions on the hearing health journey, and the emotional aspects of everyday and social life.

<u>Self-Efficacy</u>
Self-efficacy refers to the belief in ones' ability to successfully manage hearing related challenges and hearing solutions. This results in a feeling of confidence and capability.

<u>Control</u>
Control refers to a sense of power to influence and manage hearing-related challenges and hearing solutions in everyday life.

When you now translate EmpAQ-15 from English to Swedish, we ask you:

- to translate instructions, questionnaire items and scales in the attached word documents
- to focus on maintaining the conceptual meaning rather than literally translating the words in each question
- to use everyday (rather than technical) language so that the questions can be understood by everyone
- to estimate for each question (in the attached table) how difficult you felt it was to translate the question (1= not difficult at all to 10= extremely difficult) and to write the reason for your assessment in the "comment" column, if possible.

APPENDIX 1: Note that the blue text in this document was originally presented in Swedish. An online translation was performed solely to ensure accessibility for reviewers and readers.

Item	How difficult was it to translate the question? (Write a number 1-10)	Comment
1		
2		
3		
4		
5		
6		
7		
8		
9		
10		
11		
12		
13		
14		
15		

APPENDIX 1: Note that the blue text in this document was originally presented in Swedish. An online translation was performed solely to ensure accessibility for reviewers and readers.

References

Elgán, C. F., B. (2014). *Vuxet vardagsliv*: Studentlitteratur: Lund.

Gotowiec, S., Larsson, J., Incerti, P., Young, T., Smeds, K., Wolters, F., . . . Ferguson, M. (2022). Understanding patient empowerment along the hearing health journey. *Int J Audiol, 61*(2), 148-158. doi:https://doi.org/10.1080/14992027.2021.1915509

McAllister, M., Dunn, G., Payne, K., Davies, L., & Todd, C. (2012). Patient empowerment: the need to consider it as a measurable patient-reported outcome for chronic conditions. *BMC health services research, 12*(1), 157.

Wallerstein, N. (2006). What is the evidence on effectiveness of empowerment to improve health?

Zimmerman, M. A. (1995). Psychological empowerment: Issues and illustrations. *American journal of community psychology, 23*(5), 581-599.

APPENDIX 1: Note that the blue text in this document was originally presented in Swedish. An online translation was performed solely to ensure accessibility for reviewers and readers.
