# Supplementary material for: Swedish translation and content evaluation of the Empowerment Audiology Questionnaire (EmpAQ—15)
Source: J Patient Rep Outcomes. 2024 Dec 17;8:143. doi: 10.1186/s41687-024-00819-4 (PMC11652430; doi:10.1186/s41687-024-00819-4)
Supplement: Supplementary file 2 — Supplementary Material 2 [file 41687_2024_819_MOESM2_ESM.pdf]

Interview guide, Cognitive debriefing. Swedish translation of the Empowerment Audiology Questionnaire, EmpAQ

As I said before, we have translated the Empowerment Audiology Questionnaire (EmpAQ). Part of the translation procedure involves the fact that it is important to understand whether the translation you have made works for the Swedish target group. By works, I mean that the questions can be understood and that they are relevant to the group for which the questionnaire is intended.

During this interview we will go through the questionnaire step by step. When we do that, we will use a method called the "think-aloud" method. This means that you will get to read and then verbally reason about the instructions and about the questionnaire items as you answer them.

The questions consist of several statements, and we are interested in how you understand and react to them. This means that what we are primarily interested in is your feedback on the questions and not your specific answers to the various questions. There are no right or wrong answers, it is your thoughts, reactions, and musings that we are looking for.

Before we start, we will practice the technique and I will give examples of how it can be done.

For example: I know how to buy a liter of milk at my grocery store. *(Test leader goes first)*

Strongly disagree - disagree - agree – strongly agree. Not applicable.

If you were to do the same now….

Do you have any questions?

I will ask follow-up questions while you fill out the survey. After you have gone through all the statements, I will ask some additional questions about the questionnaire in general.

You are welcome to point out if there are statements that are very clear or relevant, but also if there are statements that you do not like or that do not make sense. We are interested in all your opinions, both positive and negative.

Do you have any questions before we begin?

Then we'll start right away. I will record the audio during the interview so that we have the opportunity to compile the results afterwards.

[TURN ON RECORDING EQUIPMENT]

I'm sitting here with test participant xxx and it's [today's date]

Please start by silently reading through the information on the first page.
1. Can you tell in your own words what is said in the text?
2. Can you describe if there were any difficulties in understanding the text?
3. Is there anything that you feel you would change to make it clearer?

APPENDIX 2: Note that the blue text in this document was originally presented in Swedish. An online translation was performed solely to ensure accessibility for reviewers and readers.

2023-02-22

Feel free to refer back to the information and instructions if needed as you go through the questionnaire.

Now I would like you to fill out the questionnaire. We're going to go through it question by question and I'd like you to read each question out loud and then tell me how you're thinking when you answer it. And I remind you that we want you to be as honest as you can. Both negative and positive feedback is appreciated.

1. **Item 1 [Written in Swedish]**
2. **Item 2 [Written in Swedish]**
3. **Item 3 [Written in Swedish]**
4. **Item 4 [Written in Swedish]**

Strongly disagree - disagree - agree – strongly agree. Not applicable.

**Were there any questions that were particularly difficult to understand?**	Can you tell me more about it?<br>What made it difficult?<br>Do you have any thoughts on how to formulate the statement so that it would be easier/clearer to understand?
**Were there any items that you felt were particularly relevant to you?**	Can you tell me more about it?
**Were there any questions that were not relevant to you?**	Can you tell me more about it?

5. **Item 5 [Written in Swedish]**
6. **Item 6 [Written in Swedish]**
7. **Item 7 [Written in Swedish]**

Strongly disagree - disagree - agree – strongly agree. Not applicable.

**Were there any questions that were particularly difficult to understand?**	Can you tell me more about it?<br>What made it difficult?<br>Do you have any thoughts on how to formulate the statement so that it would be easier/clearer to understand?
**Were there any items that you felt were particularly relevant to you?**	Can you tell me more about it?
**Were there any questions that were not relevant to you?**	Can you tell me more about it?

8. **Item 8 [Written in Swedish]**
9. **Item 9 [Written in Swedish]**

Strongly disagree - disagree - agree – strongly agree. Not applicable.

**Were there any questions that were particularly difficult to understand?**	Can you tell me more about it?<br>What made it difficult?<br>Do you have any thoughts on how to formulate the statement so that it would be easier/clearer to understand?
**Were there any items that you felt were particularly relevant to you?**	Can you tell me more about it?
**Were there any questions that were not relevant to you?**	Can you tell me more about it?

10. **Item 10 [Written in Swedish]**
11. **Item 11 [Written in Swedish]**
12. **Item 12 [Written in Swedish]**

Strongly disagree - disagree - agree – strongly agree. Not applicable.

**Were there any questions that were particularly difficult to understand?**	Can you tell me more about it?<br>What made it difficult?

	Do you have any thoughts on how to formulate the statement so that it would be easier/clearer to understand?
**Were there any items that you felt were particularly relevant to you?**	Can you tell me more about it?
**Were there any questions that were not relevant to you?**	Can you tell me more about it?

13. **Item 13 [Written in Swedish]**

14. **Item 14 [Written in Swedish]**

15. **Item 15 [Written in Swedish]**

Strongly disagree - disagree - agree – strongly agree. Not applicable.

**Were there any questions that were particularly difficult to understand?**	Can you tell me more about it?<br>What made it difficult?<br>Do you have any thoughts on how to formulate the statement so that it would be easier/clearer to understand?
**Were there any items that you felt were particularly relevant to you?**	Can you tell me more about it?
**Were there any questions that were not relevant to you?**	Can you tell me more about it?

Thank you!

**What do you think of the questionnaire in general?**	What did you like?<br>What didn't you like?
**What do you think about the answering scale and the answering options?**	
**What do you think about the length of the questionnaire?**	

**Were there any questions that were particularly difficult to understand?**	Can you tell me more about that?<br>What made it difficult?<br>Do you have any thoughts on how to formulate the statement so that it would be easier to understand?
[answering alternatives]	Did you find them difficult to understand?<br>How did you reason when you used the different answer options?
[statement]	If you were to use your own words, how would you explain what this statement means?<br>In what way was this statement relevant to you?
[statement]	I noticed that [X] was difficult for you to answer. Can you tell me more about it?
**Were there any issues that you felt were particularly relevant to you?**	Can you tell me more about that?
**Were there any questions that were not relevant to you?**	Can you tell me more about that?
**Were there any questions that you reacted negatively to? (was upsetting)**	Were there statements you didn't like? Why?<br>Were there any questions that you thought were too personal?<br>Were there statements that you found offensive?
**Is there something that you are missing in the questionnaire?**	Can you tell me more about that?

Then I would like to thank you for your time and your opinions.
